# Concurrent AIV as a method to hard tailor test- and model philosophies in times of need

**DOI:** 10.1016/j.mex.2019.08.010

**Published:** 2019-09-06

**Authors:** Christian Grimm, Jeffrey Hendrikse

**Affiliations:** a German Aerospace Center (DLR), Germany; b Airbus Defense and Space, Germany

**Keywords:** Concurrent AIV (C-AIV), Hayabusa2/MASCOT, Asteroid lander, Satellite design and manufacturing, C/D-phase, Concurrent AIV

## Abstract

MASCOT, a small 11 kg prototype Asteroid Lander on-board JAXA’s Hayabusa2 space probe, was launched on December 3rd, 2014, and arrived at its destined target asteroid (162173) Ryugu on June 27, 2018. MASCOT was separated from its mother spacecraft and successfully landed on October 3rd, 2018, accomplishing the first ever landing of a European spacecraft on the surface of an asteroid. To catch this particular launch opportunity its development timeline needed to be heavily compressed. In particular, the kick-off for hardware production was released in February 2012, only 2 years before the initially planned delivery of the flight unit. Due to this compact schedule, current and well established verification processes could not be followed in order to finalize the project in the given time. But by applying a unique mix of conventional and tailored model philosophies it was possible to dynamical adapt the test program to accomplish for the shortest planning and a suitable weighing of costs and risks. A strategy of Concurrent Assembly, Integration and Verification (C-AIV) helped to identify and mitigate design and manufacturing issues and shortened the test timeline further from a general 4–5 year C/D-phase down to 2,5 year C/D-phase. This short article outlines the general idea of the applied method which could be used by AIV and System Engineers in a general tailoring process for projects of similar nature which could be run in an alternative and much faster way, if the circumstances would call for it.

• Concurrent AIV, a new agile methodology to hard tailor test and model philosophies for space projects is presented,
• The methodology is based on parallelization of test activities, creation of independent unique test threads and synergizing their dependencies at key points,
• On the baseline of the asteroid lander MASCOT, this methodology has been successfully applied to shorten the overall test and implementation schedule to only 2.5 years.

**Specification Table**

**Table:** 

Subject area	Engineering
More specific subject area:	Complex Systems Engineering Design and Manufacturing of Space Systems
Method name:	Concurrent AIV (C-AIV)
Name and reference of original method:	- Assembly, Integration and Verification (AIV), E.g.: ECSS, Space Engineering - Verification Guidelines - ECSS-E-HB-10-02A, ESA Requirements 1180 and Standards Division (2010). - Concurrent Engineering, E.g.: https://www.dlr.de/irs/en/desktopdefault.aspx/tabid-11079/#gallery/27740 - Lean Manufacturing E.g.: S. Shahbazi, S. Javadi, Supporting Production System Development Through Obeya Concept, Lap Lambert Academic Publishing, 2015.
Resource availability:	NA

## Method details

**Fig. 1:**
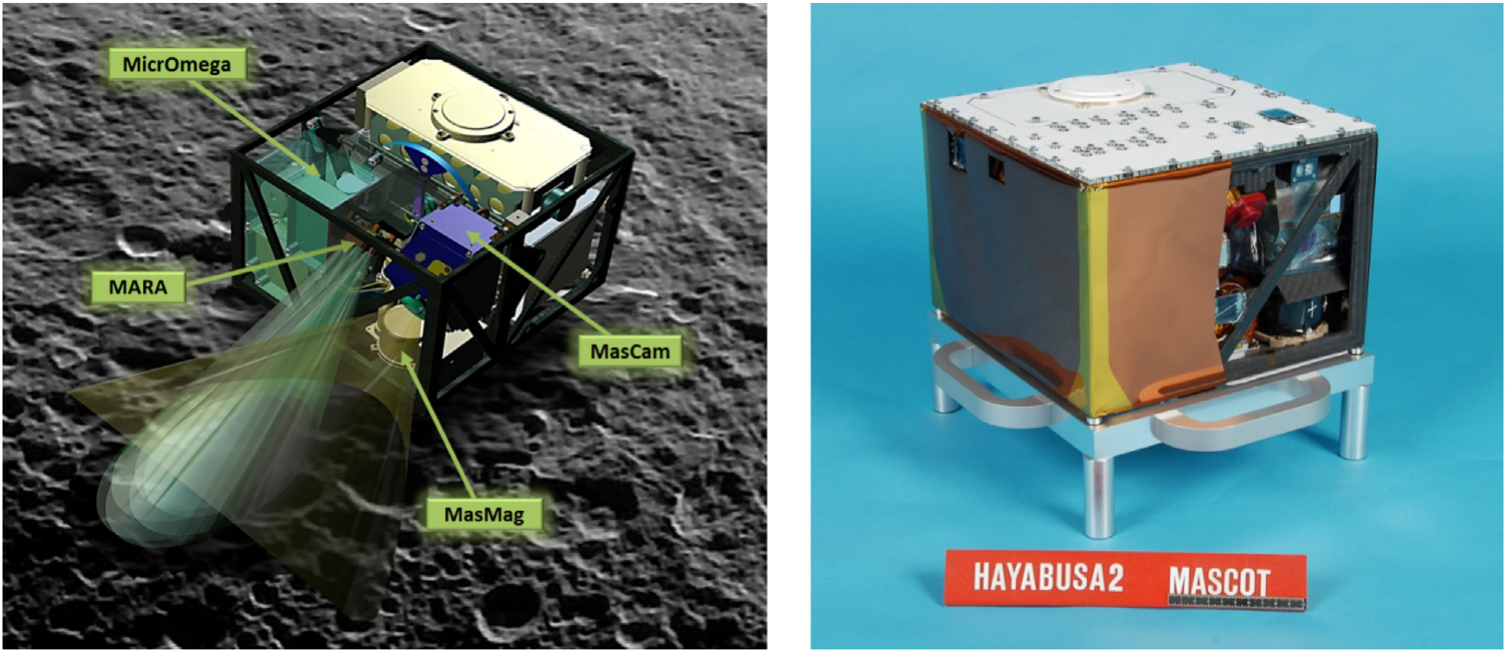
Left: Artists impression of the landed MASCOT on the surface of 162173 Ryugu indicating the operation of its four payloads; Camera (MasCAM), Radiometer (MARA), Magnetometer (MasMAG) and Infrared Microscope (MicrOmega). Right: MASCOT Flight Model (landing module only) before attachment to Hayabusa2.

**Fig. 2:**
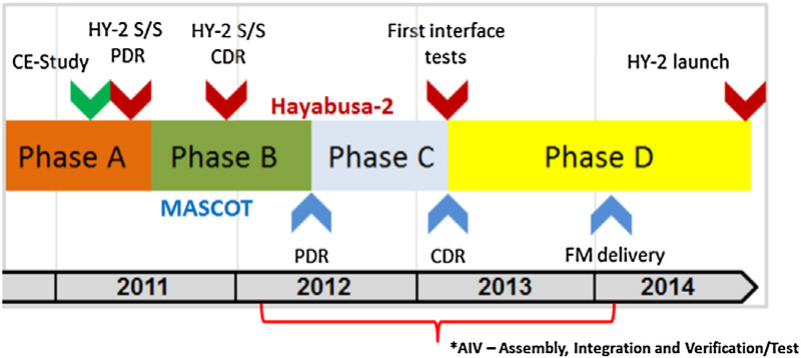
MASCOT project timeline with major milestones.

As today’s projects increase quickly in complexity and development times are shortened to save budgets, schedules become so compressed, and resources are so constrained, that the corporate goal of such projects is to overcome impossible odds and to achieve miracles [1]. The DLR MASCOT project, a small 11 kg Asteroid landing package on-board JAXA’s Hayabusa2 space probe launched on December 3rd, 2014, had such constraints (Figs. 1 and 2 ). Selected at a time when its conceptual design and scientific payloads had not been fully defined; with the carrier spacecraft already in its critical design phase having most of its interfaces fixed; only 2 years left until a proposed final delivery of the flight unit; and no heritage to use off-the-shelf equipment directly, a full prototype design of a miniaturized asteroid lander to an unknown target became necessary [2,3].

The typical life cycle model for space projects as defined by ESA and NASA is divided in 6–7 phases. These phases stem from the original partition of lifecycle stages as defined in the ISO/IEC 15288: Concept Stage (0/A), Development Stage (A/B), Production Stage (C/D), Utilization and Support Stage (E), Retirement Phase (F) [4].

The first phase 0/A, derives appropriate mission requirements starting from the mission statement and mission objectives, including a stakeholder analysis, through Functional Analysis and Concept of Operations [11]. The next phase A/B, is dedicated to detailing and improving the design through first hardware breadboard tests and a preliminary design analysis. Phases C and D are then dealing with the implementation and realization of the system. This typically involves extensive Assembly, Integration and Verification (AIV) activities which include the simulation of and test under the expected space environment and flight operation to verify and demonstrate the overall performance and reliability of the flight system. From the beginning and valid for all phases, choosing the right model philosophy or approach of the Verification and Validation (V&V) process is crucial and driven by risk tolerance. Less verification implies but does not necessarily create more risk. More verification implies but does not guarantee less risk [5].

In European and American space industry there are currently two main model philosophies in use to conduct the verification of a space system. These two philosophies are known as the Prototype Approach, sometimes also called the Traditional or Classical Approach, and the Protoflight Approach [[5], [6], [7]]. The basic difference is reflected in the number and types of models being built and tested. In the Classical Approach the design verification evolves in a mostly sequential and also successive fashion from a Breadboard model (BB), a Structural or Structural-Thermal Model (SM or STM), an Electrical Model (EM), a Qualification or Engineering Qualification Model (QM or EQM), to the final Flight Model (FM), which may also have a sister model used as Flight Spare (FS) in case of launch failure or otherwise as Ground Reference Model (GRM). The Protoflight Approach qualifies the design of a single flight model by replacing critical subsystems during the integration process. The Protoflight Model (PFM) is subject to a full qualification process and is refurbished before launch. It is generally faster and cheaper and is applied to projects with no technology critical design accepting a medium risk.

The Classical Approach would be of course the most reliable method to choose as it gives the highest confidence that the final product performs well in all aspects of the mission. However, due to the tight schedule in the MASCOT project, the extensive and time consuming method of this approach could not be applied. On the other hand, the Protoflight Approach was also not applicable, since the chosen payloads and the system itself had very heterogeneous maturity levels, which prevented the system from being tested as a consistent entity at each stage. Hence, the test philosophy of MASCOT applied a Hybrid Approach with a mixture of conventional and tailored model strategies. This approach is common practice in scientific robotic missions [5] but the specific MASCOT model philosophy went even further (Fig. 6). The project started with a baseline on the Classical Approach (STM, EQM and FM) to ensure a minimum number of physical models required to achieve confidence in the product verification with the shortest planning and a suitable weighing of costs and risks. But this approach was adapted on a case by case scenario, where the model philosophy evolved along the verification and test process depending on the particular system and subsystem readiness. According to this dynamical process, the decision which model to test and what to test with it was often made simply on the subsystems availability (Fig. 7). This included test models reorganization, refurbishing and re-assigning previous models for other verification tasks if appropriate, skipping test cases, parallel testing of similar or equal models and for some components allowing the qualification on MASCOT system level (Table 1).

**Table 1:** MASCOT System Level Hierarchy.

1	Hayabusa2 Spacecraft Level
2	MASCOT System Level
3	MASCOT Module Level
4	MASCOT Equipment Level
5	Single Component Level

**Fig. 3:**
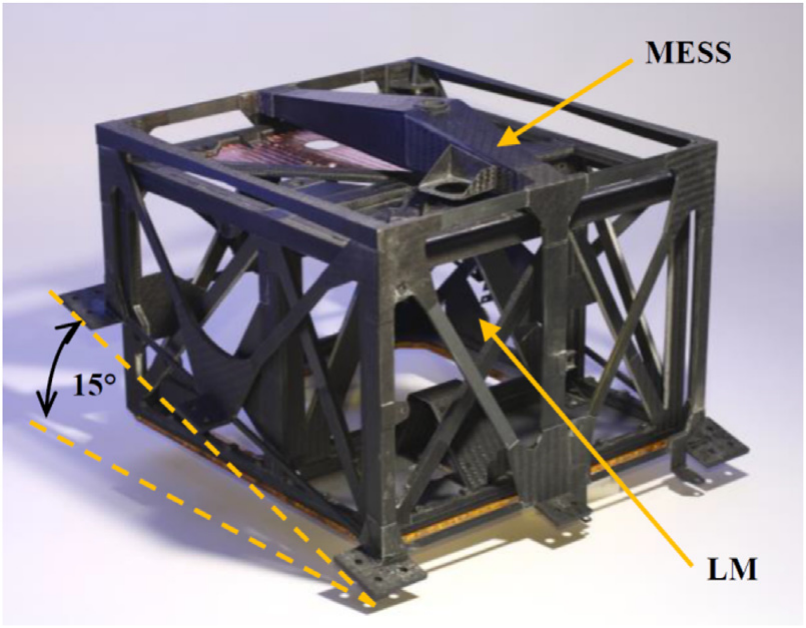
MASCOT LM and MESS structure.

**Fig. 4:**
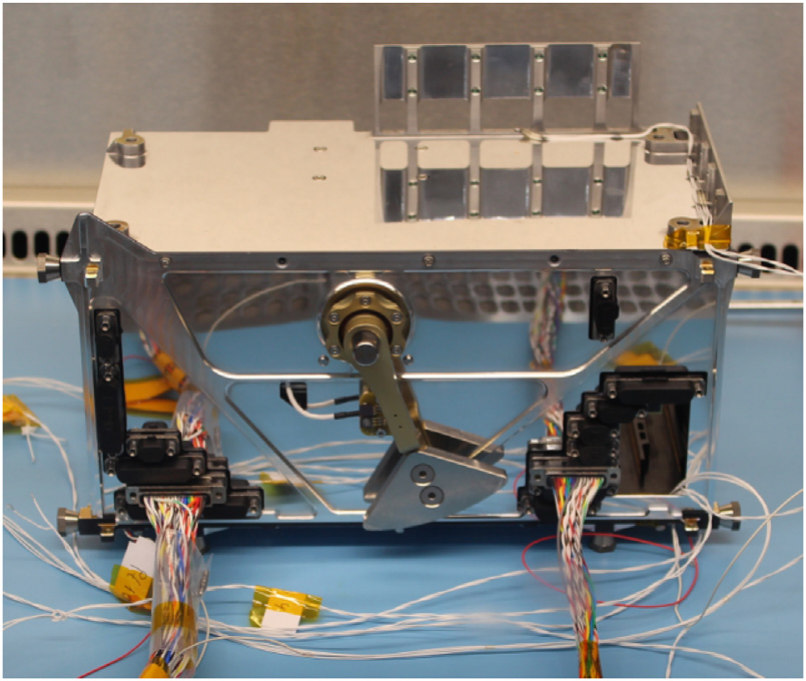
MASCOT Ebox.

The verification approach was focused around the systems primary structure elements (Fig. 3, Fig. 4). The frame structure comprises of the MASCOT Landing Module (LM), the Mechanical and Electronic Support System (MESS), which is the main interface to HY-2 remaining at the spacecraft after separation, and the common electronic box (Ebox), which is an integral part of the LM structure serving also as interface for other subsystems like the mobility unit, the battery and the communication modules. The development status of these three elements defined the overall maturity of each MASCOT model.

The heterogeneous maturity levels of the other sub-systems have let to tailor a mixed model philosophy into an adaptable overall MASCOT system strategy to maintain reduced programmatic risks. Due to the highly compact and lightweight nature of this system almost all elements were custom made for the specific mission scenario. The risk assessment showed that a high chance for schedule delays could occur due to test repetition of unit failures or late delivery. Keeping this course, the complete path would have taken approximately 48 month. However, when your ride has minimal options to wait for you defining a time limit less than 24 month and none of the subunits are replaceable by off-the-shelf equipment, how do you proceed?

**Fig. 5:**
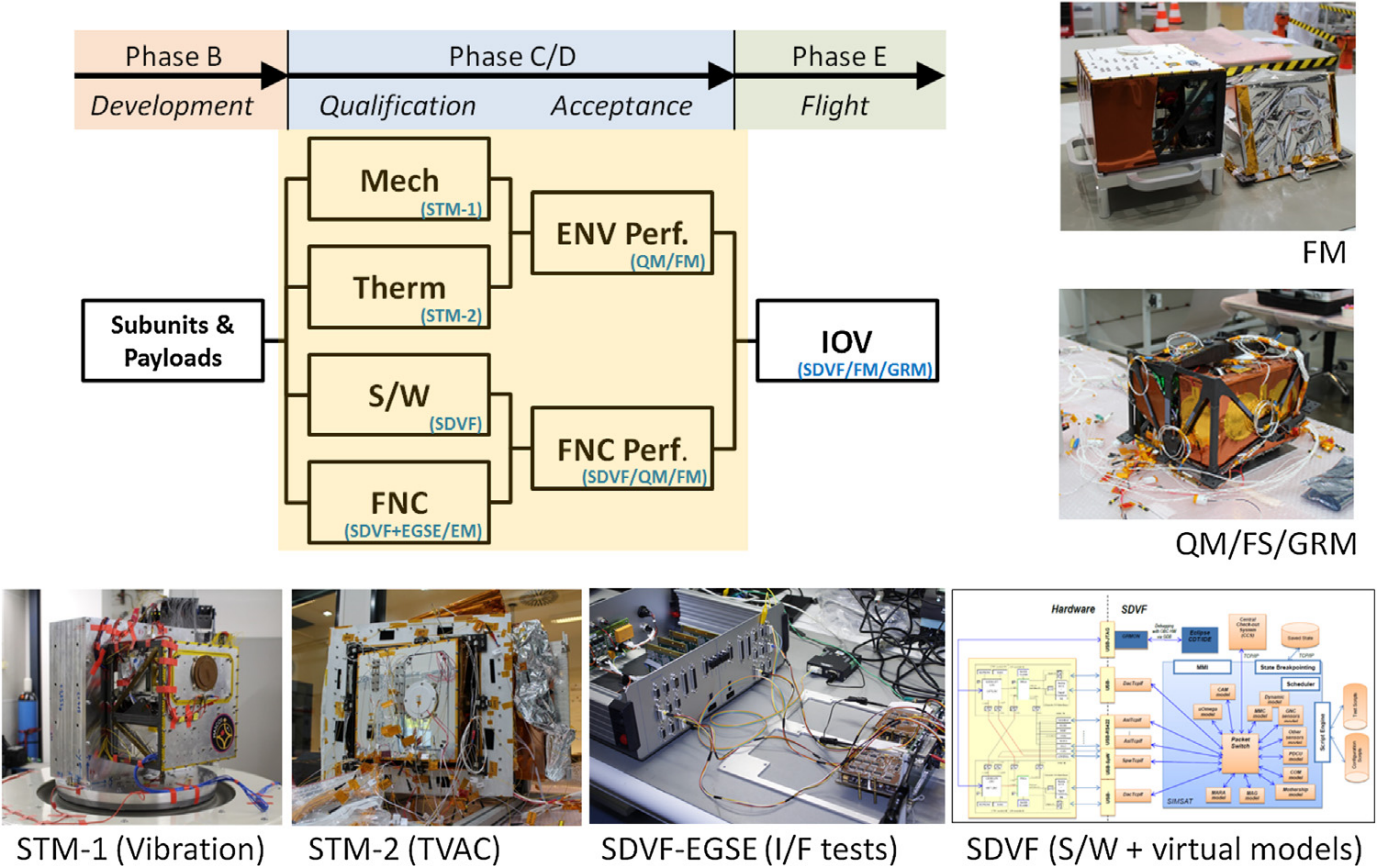
MASCOT Concurrent AIV (C-AIV) framework. Parallelization of test activates for Mechanical and Thermal Testing, Software and Functional Testing and later for Environmental and Functional Performance Testing as well as In-Orbit Verification after launch.

To catch up with the HY-2 development schedule and maintain enough margins to incorporate risk, the MASCOT project incorporated parallelization of testing activities using identical copies of the primary structure elements and flexibility in the shared model philosophy. This in turn created independent unique test threads only joining their dependencies at key points where optional other roads could be chosen. In example, if the first test model was damaged by one test the second was shortly available to redo the test if applicable. With these near parallel development lines the precious project time-line could be adjusted more freely and with it forestalling a potential delay due to an additional 4 months + manufacturing process. Like Concurrent Engineering, a methodology based on the parallelization of engineering tasks nowadays used for optimizing and shorten design cycles in early project phases, we introduce here the term “Concurrent AIV (C-AIV)” to express the many simultaneous running test and verification activities (Fig. 5).

**Fig. 6:**
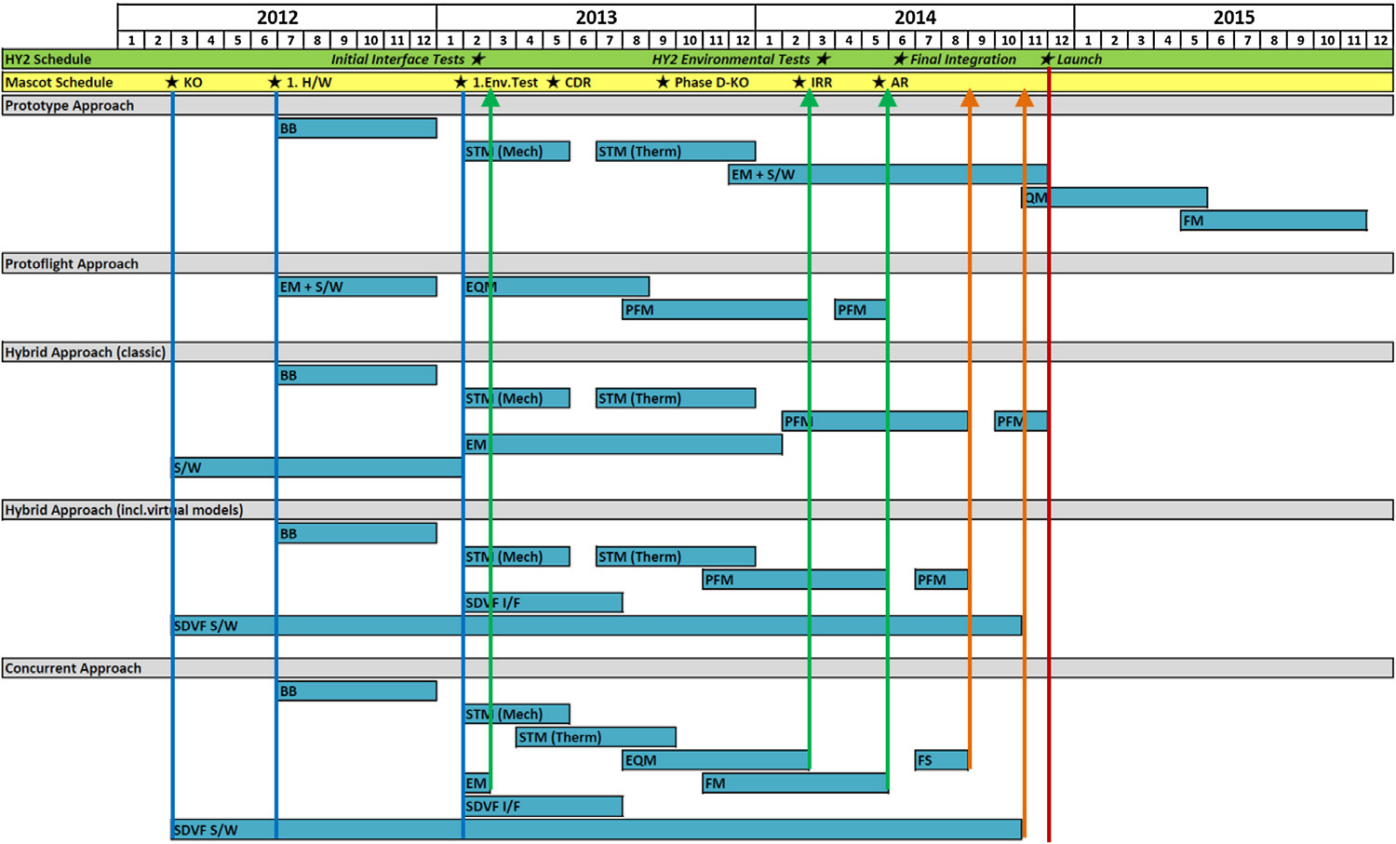
Comparison of different verification approaches. At the top are the main schedules of the MASCOT lander as well as the main spacecraft Hayabusa2, including the kick-off (KO) and other milestone. One can see that with a normal classical Prototype approach using sequential models and test it is not feasible to manage the full program before launch. The protoflight approach is only possible, if one can build on already developed hard- and software which can be refurbish just before launch. But if the system and specifically the subsystems are in very heterogeneous maturity levels this doesn’t work. Very often a good compromise between these two is a combination, a classical hybrid approach, having an early environmental qualification program on STM levels and in addition EM units in parallel where the S/W of the system could be tested. This could be squeezed together when one is using virtual models. Since simulation technology is already quite advanced, running the S/W on virtual models before implementing it on real H/W helps minimizing I/F problems and possible delays later during integration and testing. But it would still have a high risk; if for example, during testing the single model gets damaged. Nor would it be possible to deliver earlier models if required by the main spacecraft. As it was required in this project at certain points (green arrows), to take part in the initial interface tests as well as for the acceptance test on main spacecraft level. As a consequence, one can take the multi-model approach, parallelize them and squeeze them to the extent that one remains in the given time and being able to perform all necessary qualification and acceptance tests. In addition, one manages also to bring-in in time the refurbished EQM as FS and update the S/W (orange arrows) to the last most reliable version at the S/C last hard-wired connection on ground before it is put on the launch adapter.

**Fig. 7:**
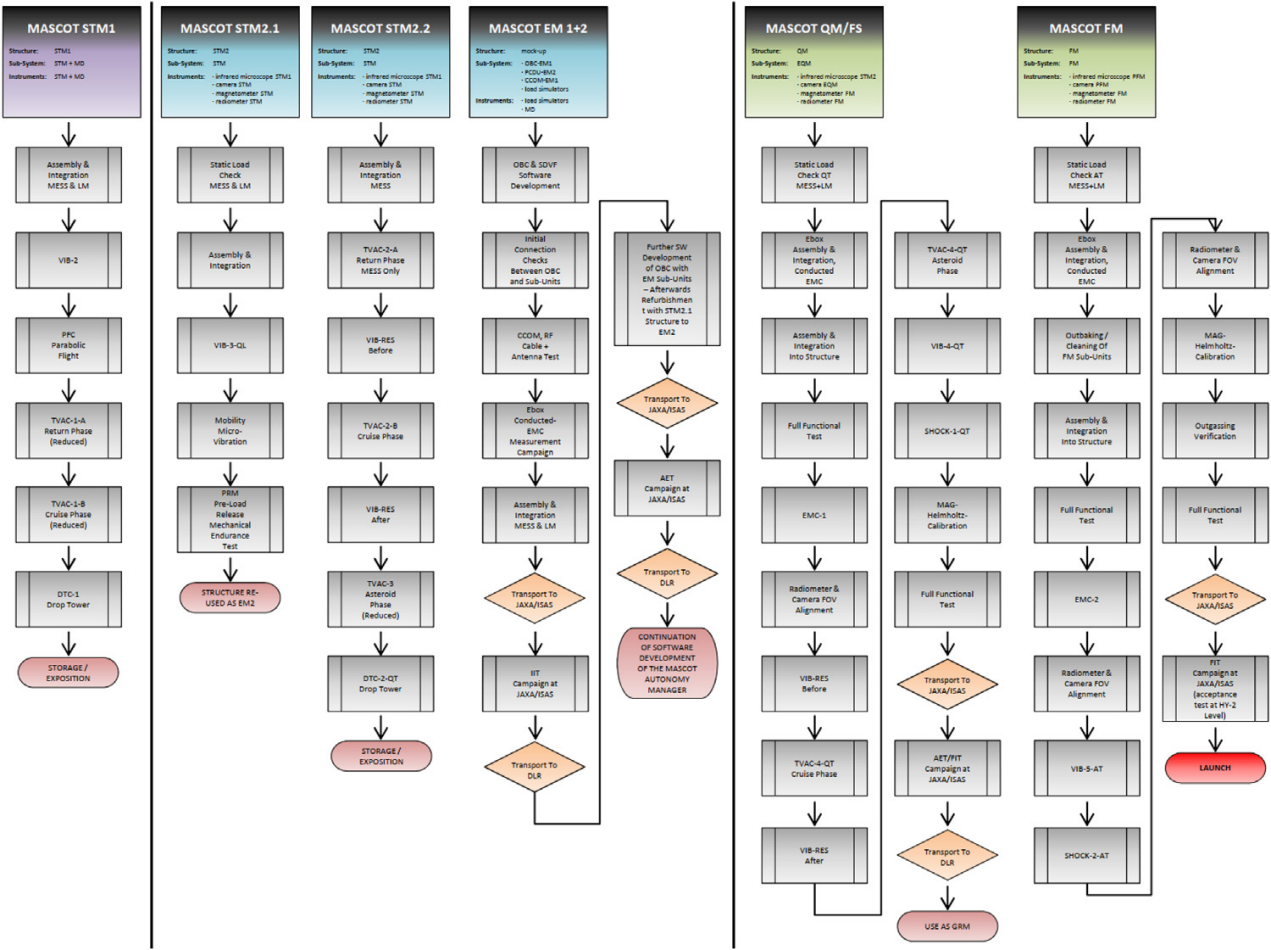
Full model work flow of the individual MASCOT models, showing their testing sequence, reorganization and final verification.

In effect, the development, test and verification track of Software Development, Functional Performance, Mechanical- and Thermal Verification got their own independent test routes sharing their verification processes. Also, certain flexibility between these 4 major threads allowed for in-parallel subunit testing. Almost all environmental and functional tests with subsystems could be performed on EM and STM level before the EQM and FM were fully assembled which effectively reduced potential delays. In addition, both these final threads (EQM/FM) performed in near parallel activities shared again their verification processes. The EQM endured all environmental qualification tests herewith validating parts of the FM which in turn did its final mechanical and electrical acceptance tests on HY-2 system level, hereby reducing again required project timeline. This approach also reduced testing stress on the potentially flying models which in the conventional approach would have undergone full acceptance testing on MASCOT level as well as HY-2 level.

The challenges in creating parallel development lines were found mainly in team and facility resources as these were not always readily and on-demand available. This philosophy was also more complex as it requires the overview of the development process of the mother spacecraft, the ongoing progress on system level as well as the insight in all payloads and subsystems. This was handled by splitting the tasks on more Systems Engineering and AIV responsible personnel and performing regular consolidation gatherings between these key players including also the Project Management and Product Assurance, in order to keep the project sorted and on course. In addition, Obeya meetings were held daily, strictly limited in time and based mainly on current test schedules and observed non-conformances [8,9]. This allowed the core team to quickly react on critical matters saving valuable time usually lost easily in hierarchy driven management decision processes.

Overall, with the applied strategy and method, the team has successfully completed approximately 30 MASCOT System and Module Level tests, more than 50 additional Equipment Level tests (excluding payloads) as well as approximately 10 test campaigns on its carrier spacecraft Hayabusa2. This culminates in almost 100 different test campaigns performed in roughly half the time allocated for such a prototype project which would have followed a standardized way.

In Summary:

**Table:** 

PRO	CON
• Compress a normal 4 year AIV phase into 2,5 years! (Mascot case ∼100 Tests in 2 years), • Multiple models/spares, • High rate of flexibility (re-use models for other tests), • Test early/Test often, • Robust system, focus on what is really necessary, (requirements can be changed, test limits are adaptable), • Core team decisions (no hierarchy), • Solve issues in parallel with possibility to implement late changes (no-frozen design concept), • Lean documentation, Incorporation of tools from the Toyota Production System (e.g. Obeya, short and objectives driven meetings),	• Complex multi-layer approach, • Subsystem development/qualification also in parallel and in some cases on system level, • Highly resource demanding (personnel, facilities), • Heavy work load for project team and partners, • Documentation: no common style, not always completely reviewed before test, • Late awareness of test results (outcome of detailed analysis), • High risk to miss something, • Proper maturity only at FM, • Intense late access activities, • Some tests need to be skipped or performed in-orbit during cruise,

## Conclusion

According to the standards currently in use, like the one from the European Cooperation for Space Standardization (ECSS) or from the NASA Technical Standards Program (NTSP), such a plan would have been classified as impossible and would have been cancelled due to lack of available schedule time. However, as performed and shown in the MASCOT project an alternative answer would be to leave the comfortable zone of the known standards, reiterate the given requirements and establish a hard-tailored minimum standard which achieves both, enough confidence in the products performance as well as finding the shortest planning including a suitable weighing of costs and risks.

In addition, to perform such an “agile” approach took some reorientations in the normally applied and used to “absolute-minimized-risk” oriented verification ideology. Those adjustments needed to be but were not limited to:

• Common sense and engineering experience as a driver for quick decisions inside the core team;
• Allowing the communication of experts (even from different organizations) directly between each other with no hierarchy implied bottlenecks;
• Lean documentation with no formal document style and no extensive signature loops;
• Including subcontractors as project partners to understand the need to implement small changes even at later stages;
• Quality Assurance is not only a control entity, but builds the interface to established processes and guidelines, but in a way that these can be adjusted where applicable.
• Avoiding blame culture and practicing problem solving culture

For a full review of this method and a historical comparison to other fast-paced projects and programs in space showing large similarities to Skunk Works, the 1960s Space Race and Faster Better Cheaper, please refer to [10].
