# Supplementary figures and images for: Concurrent AIV as a method to hard tailor test- and model philosophies in times of need
Source: MethodsX. 2019 Sep 6;6:2148–55. doi: 10.1016/j.mex.2019.08.010 (PMC6812338; doi:10.1016/j.mex.2019.08.010)

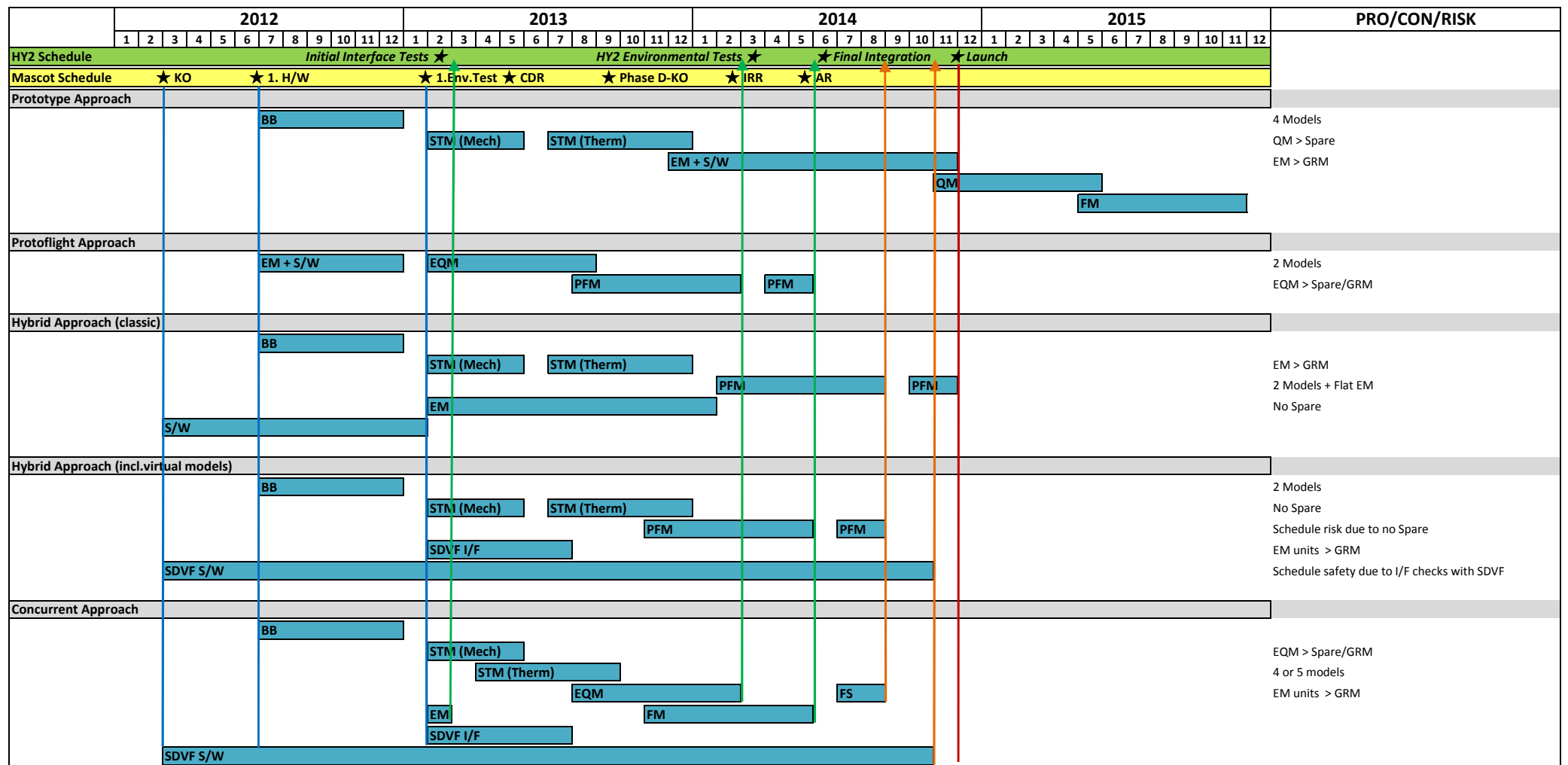

Supplement: Supplementary file 1 [file mmc1.pdf]
